# Upregulation of ZIP14 and Altered Zinc Homeostasis in Muscles in Pancreatic Cancer Cachexia

**DOI:** 10.3390/cancers12010003

**Published:** 2019-12-18

**Authors:** Ahmad Rushdi Shakri, Timothy James Zhong, Wanchao Ma, Courtney Coker, Sean Kim, Stephanie Calluori, Hanna Scholze, Matthias Szabolcs, Thomas Caffrey, Paul M. Grandgenett, Michael A. Hollingsworth, Kurenai Tanji, Michael D. Kluger, George Miller, Anup Kumar Biswas, Swarnali Acharyya

**Affiliations:** 1 Institute for Cancer Genetics, Columbia University Irving Medical Center, New York, NY 10032, USA; as5797@cumc.columbia.edu (A.R.S.); tjz2104@cumc.columbia.edu (T.J.Z.); wm49@cumc.columbia.edu (W.M.); cc3703@cumc.columbia.edu (C.C.); akb2180@cumc.columbia.edu (A.K.B.); 2 Graduate School of Arts and Sciences, Department of Pathobiology and Mechanisms of Disease, Columbia University Irving Medical Center, New York, NY 10032, USA; 3 Department of Biological Sciences, Columbia University, New York, NY 10027, USA; sk4251@columbia.edu; 4 Barnard College, Columbia University, New York, NY 10027, USA; Sdc2157@barnard.edu (S.C.); hrs4001@med.cornell.edu (H.S.); 5 Department of Pediatrics, Weill Cornell Medicine, Cornell University, New York, NY 10065, USA; 6 Department of Pathology and Cell Biology, Columbia University Irving Medical Center, New York, NY 10032, USA; mjs59@cumc.columbia.edu; 7 Eppley Institute for Research in Cancer and Allied Diseases, Fred & Pamela Buffett Cancer Center, University of Nebraska Medical Center, Omaha, NE 68198, USA; thomas.caffrey@unmc.edu (T.C.); pgrandgenett@unmc.edu (P.M.G.); mahollin@unmc.edu (M.A.H.); 8 Division of Neuropathology, Department of Pathology and Cell Biology, Columbia University Irving Medical Center, New York, NY 10032, USA; kt8@cumc.columbia.edu; 9 Department of Surgery, Columbia University Irving Medical Center, New York, NY 10032, USA; Mk2462@cumc.columbia.edu; 10 Department of Surgery, New York University Grossman School of Medicine, New York, NY 10016, USA; george.miller@nyumc.org; 11 Department of Cell Biology, New York University Grossman School of Medicine, New York, NY 10016, USA; 12 Herbert Irving Comprehensive Cancer Center, Columbia University Irving Medical Center, New York, NY 10032, USA

**Keywords:** cachexia, ZIP14, *Slc39a14*, pancreatic cancer, pancreatic ductal adenocarcinoma, zinc transporter, zinc homeostasis, metastasis, muscle atrophy

## Abstract

Pancreatic ductal adenocarcinoma (PDAC) is a lethal cancer type in which the mortality rate approaches the incidence rate. More than 85% of PDAC patients experience a profound loss of muscle mass and function, known as cachexia. PDAC patients with this condition suffer from decreased tolerance to anti-cancer therapies and often succumb to premature death due to respiratory and cardiac muscle wasting. Yet, there are no approved therapies available to alleviate cachexia. We previously found that upregulation of the metal ion transporter, *Zip14*, and altered zinc homeostasis are critical mediators of cachexia in metastatic colon, lung, and breast cancer models. Here, we show that a similar mechanism is likely driving the development of cachexia in PDAC. In two independent experimental metastasis models generated from the murine PDAC cell lines, Pan02 and FC1242, we observed aberrant *Zip14* expression and increased zinc ion levels in cachectic muscles. Moreover, in advanced PDAC patients, high levels of ZIP14 in muscles correlated with the presence of cachexia. These studies underscore the importance of altered ZIP14 function in PDAC-associated cachexia development and highlight a potential therapeutic opportunity for improving the quality of life and prolonging survival in PDAC patients.

## 1. Introduction

Pancreatic ductal adenocarcinoma (PDAC) is an aggressive cancer with a five-year survival rate of 9.3% [1,2]. Surgical resection is currently the only curative option and is only available for localized and non-metastatic PDAC [3]. However, at the time of diagnosis, 70–80% of PDAC patients present with either locally advanced (stage-III) or metastatic (stage-IV) disease, which dramatically limits their options for surgery and cure [4,5,6,7]. Strikingly, the prognosis for metastatic PDAC patients ranks among the lowest of all cancers, with a five-year survival rate of 2% and a median survival of 3–6 months with systemic standard-of-care chemotherapy treatment [8,9,10,11]. The discovery of effective treatments for patients with advanced PDAC therefore continues to be a major clinical challenge.

Cachexia is a metabolic syndrome associated with the loss of muscle mass and function, which affects 85% of PDAC patients [12]. Cancer cachexia is characterized by a decrease in both energy intake and muscle protein synthesis, coupled with an increase in both energy expenditure and muscle protein degradation [13,14,15]. During cachexia development, loss of skeletal and cardiac muscle mass and/or function is often accompanied by anorexia, chronic inflammation, and adipose tissue lipolysis [5,13,14,16]. In particular, the wasting of cardiac and skeletal muscles compromises vital physiological functions, such as circulation (heart muscles), respiration (diaphragm muscles), and locomotion (limb muscles), and thereby contributes to a poor quality of life and reduced survival for PDAC patients [12,16,17,18]. Furthermore, cachexia cannot be reversed by nutritional supplementation alone; this points to a systemic metabolic dysfunction in patients with this condition [5,14,18]. Cachectic cancer patients also exhibit a decreased tolerance and poor response to chemotherapeutic agents, which further accelerates the progression of their disease [19]. Therefore, it is imperative to understand the mechanisms driving PDAC-associated cachexia to devise effective strategies for its treatment.

Studies over the past few decades have identified several mediators of cancer cachexia; however, none of the drugs designed to target these preclinical candidates showed efficacy in clinical trials for the treatment of cancer-associated cachexia in many cancer types, including PDAC [20]. To address the challenges associated with identifying and targeting the mediators of cancer cachexia, our laboratory recently demonstrated that the metal ion transporter *Slc39a14* (*Zip14*) is upregulated in cachectic muscles in the context of metastatic colon, lung, and breast cancers. We found that aberrantly elevated expression of *Zip14* in muscles increases the levels of intracellular zinc in muscle cells and perturbs zinc homeostasis. We further showed that excess zinc in mature muscle fibers promotes muscle breakdown by inducing myosin heavy chain loss, and that increased zinc levels in muscle progenitor cells inhibit muscle–cell differentiation and prevent the compensatory formation of new muscle [21]. The *Zip14*–zinc pathway is therefore a mediator of cachexia development in metastatic colon, lung, and breast cancer models. In the current study, we investigated whether this pathway also serves as an underlying mechanism of PDAC-associated cachexia pathogenesis. We examined two independent experimental metastasis models generated from murine PDAC lines (Pan02 and FC1242) that induce severe cachexia, and found elevated *Zip14* expression and increased intracellular zinc levels in their cachectic muscles. These experimental findings were clinically validated using cachectic PDAC patient muscle samples, in which elevated ZIP14 levels in muscle were significantly associated with the presence of cachexia. Since *Zip14* is not normally expressed in healthy muscle cells, interfering with the ZIP14–zinc axis in muscle might prevent cachexia development in PDAC patients and improve their outcomes. Future preclinical and clinical studies are therefore warranted to test the efficacy of ZIP14 blockade or zinc chelation as a therapeutic strategy to prevent or reverse PDAC-associated cachexia.

## 2. Results

### 2.1. Cachexia Development in Experimental Metastatic Models of PDAC

**Figure 1:**
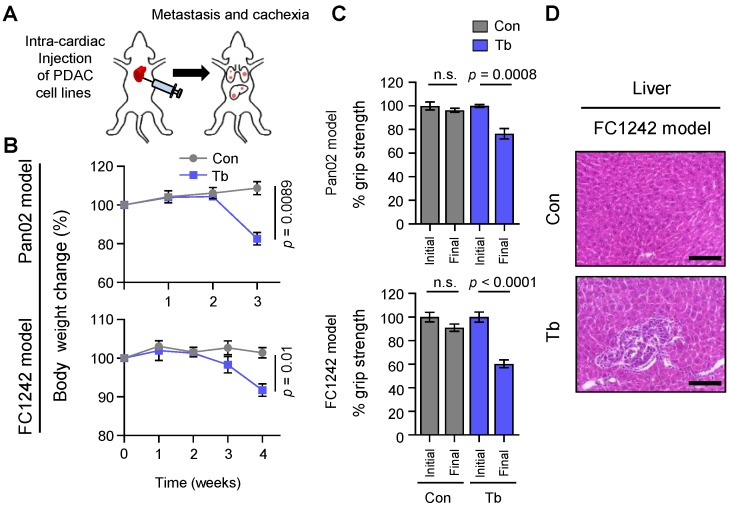

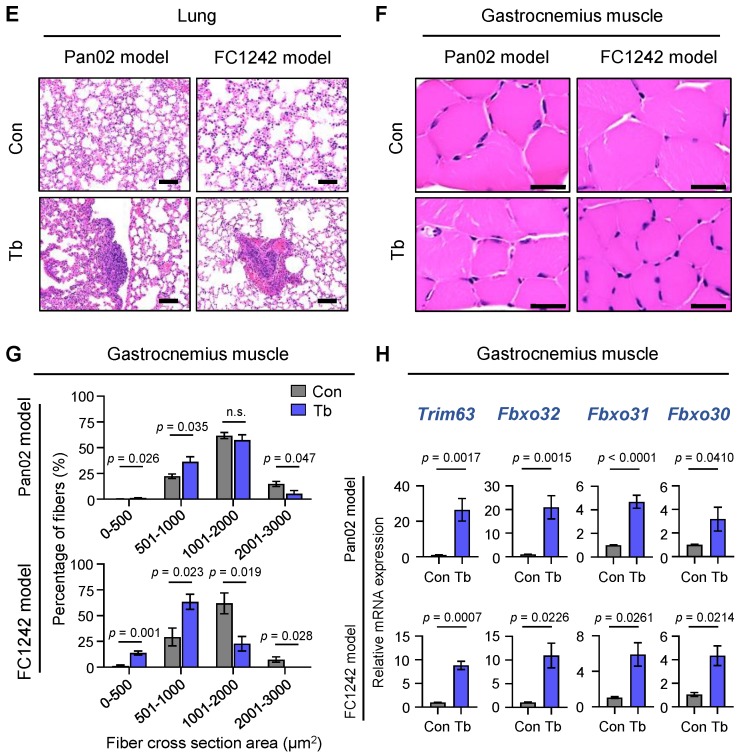
Cachexia development in experimental metastasis models of pancreatic ductal adenocarcinoma (PDAC). (**A**) Schematic illustration of intra-cardiac injection of 1 × 10^5^ Pan02 or FC1242 cells into the arterial circulation of mice and the development of metastasis. (**B**) Body weight analysis of tumor-bearing mice harboring Pan02 and FC1242 metastases (blue lines) compared to controls (gray lines). (**C**) Measurements of hind-limb grip strength in Pan02 and FC1242 tumor-bearing mice (Tb, blue bars) compared to controls (Con, gray bars) following tumor-cell injection. (**D**) Representative hematoxylin and eosin (H&E) staining of liver tissue sections from the FC1242 model compared to control. Scale bars represent 100 µm. (**E**) Representative H&E staining of lung tissue sections from Pan02 and FC1242 mice compared to controls. Scale bars represent 100 µm. (**F**) Representative H&E staining of gastrocnemius muscle cross-sections from Pan02 and FC1242 mice compared to controls. Scale bars represent 25 µm. (**G**) Quantitation of gastrocnemius muscle fiber cross-sectional area (CSA) from Pan02 and FC1242 mice compared to controls. Morphometric analysis is shown as distribution frequency of fiber CSA. (**H**) Results from real-time quantitative reverse transcription PCR (qRT-PCR) analysis of muscle atrophy markers *Trim63*, *Fbxo32*, *Fbxo31*, and *Fbxo30* in the gastrocnemius muscles from Pan02 (top) and FC1242 (bottom) mice compared to controls. *n* = 3–5 mice/group. Data are expressed as mean ± standard error of the mean (SEM). *p*-values were determined by the two-tailed, unpaired Student’s *t*-test. n.s.: Not significant; Con: Control; Tb: Tumor-bearing.

To identify mechanisms of PDAC-associated cachexia, we generated two experimental metastasis models of PDAC derived from the murine PDAC cell lines Pan02 and FC1242. These cell lines were selected to model advanced human PDAC based on three criteria: (a) The ability to metastasize, (b) the ability to induce severe cachexia [9], and (c) a mutational spectrum that is distinct between the two cell lines to rule out mutation- or cell line-specific effects. The Pan02 cell line is a metastatic, *SMAD4*-null murine PDAC cell line isolated from a primary pancreatic tumor that arose in a C57BL/6 mouse treated with the carcinogen 3-methylcholanthrene [9,22]; and the FC1242 cell line was isolated from a PDAC tumor that arose in a C57BL/6 mouse harboring common genetic drivers of PDAC (*LSL-Kras^G12D^*; *LSL-Trp53^R172H^*; *Pdx1-Cre*) [9]. We injected 1 × 10^5^ murine Pan02 and FC1242 PDAC cells into arterial circulation via intra-cardiac injection in 8- to 9-week-old athymic nude and syngeneic C57BL/6 male mice, respectively (schematic in Figure 1A). Mice injected with either Pan02 or FC1242 cell lines gradually developed metastasis and cachexia (Figure 1A–H). In particular, marked body weight loss (Figure 1B) and reduction in hind-limb grip strength (Figure 1C), which coincided with the development of liver (Figure 1D) and lung (Figure 1E) metastases, were observed in tumor-bearing mice compared to age-matched control mice. The functional deficit in grip strength was associated with a reduction in muscle size, as quantified by morphometric analysis of myofiber cross-sectional area (CSA) (Figure 1F,G). Gastrocnemius muscles from Pan02 and FC1242 tumor-bearing mice showed features of muscle atrophy (reduction in muscle size) and harbored a greater percentage of myofibers with a smaller CSA (Pan02 fiber CSA 0–500 µm^2^: *p* = 0.026 and 501–1000 µm^2^: *p* = 0.035; FC1242 fiber CSA 0–500 µm^2^: *p* = 0.001 and 501–1000 µm^2^: *p* = 0.023) and a lower percentage of myofibers with a larger CSA (Pan02 fiber CSA 2001–3000 µm^2^: *p* = 0.047; FC1242 fiber CSA 1001–2000 µm^2^: *p* = 0.019 and 2001–3000 µm^2^: *p* = 0.028) compared to control mice (Figure 1F,G). To further validate cachexia development using established molecular markers of muscle wasting, we examined the expression of genes that encode ubiquitin ligases associated with muscle proteolysis (*Trim63/MuRF1*, *Fbxo32/MAFBx*, *Fbxo31*, *Fbxo30/Musa1*). As expected, cachectic gastrocnemius muscles showed an upregulation of the E3 ligase genes in both Pan02 (*Trim63*: *p =* 0.0017; *Fbxo32*: *p =* 0.0015; *Fbxo31*: *p* < 0.0001; *Fbxo30*: *p* = 0.0410) and FC1242 (*Trim63*: *p* = 0.0007; *Fbxo32: p* = 0.0226; *Fbxo31*: *p* = 0.0261; *Fbxo30*: *p* = 0.0214) tumor-bearing mice compared to their age-matched controls (Figure 1H). These results suggest that the experimental metastasis models derived from Pan02 and FC1242 cell lines gradually develop characteristic features of cachexia development.

### 2.2. Zip14 Is Upregulated in Cachectic Gastrocnemius Muscles with Increased Zinc Levels in the Pan02- and FC2142-Derived Experimental PDAC Models

**Figure 2:**
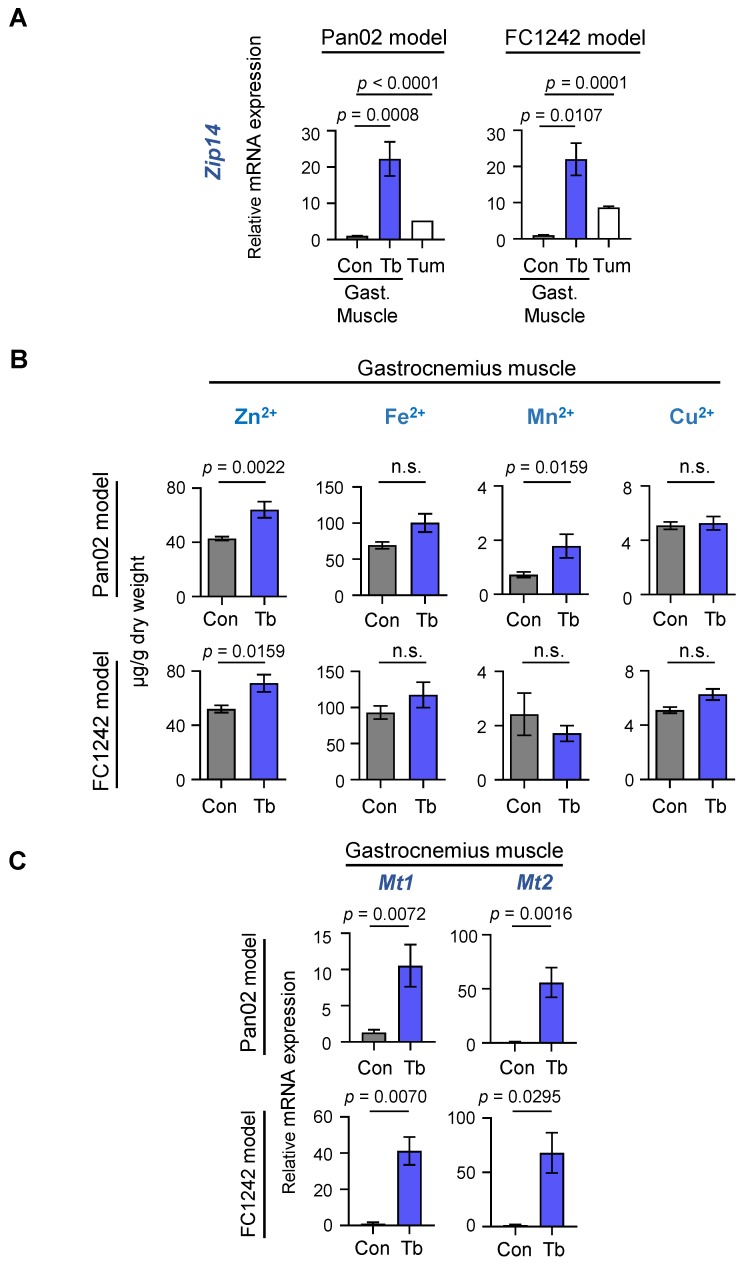
*Zip14* is induced in cachectic muscles in PDAC models and is associated with elevated zinc levels. (**A**) Results from qRT-PCR analysis of *Zip14* in the gastrocnemius (Gast.) muscles from Pan02 (left) and FC1242 (right) mice (Tb, blue bars) compared to controls (Con, gray bars) and Pan02 and FC1242 cell lines (Tum, white bars). (**B**) Results from metal ion analysis of zinc (Zn^2+^), iron (Fe^2+^), manganese (Mn^2+^), and copper (Cu^2+^) on gastrocnemius muscles from Pan02 (top) and FC1242 (bottom) models compared to control mice at endpoint. Results are depicted as micrograms (µg) of metal ion per gram (g) of muscle dry weight. (**C**) Results from qRT-PCR analysis of *Mt1* and *Mt2* in the gastrocnemius muscles from Pan02 (top) and FC1242 (bottom) mice (Tb, blue bars) compared to controls (Con, gray bars). *n* = 3–5 mice/group. Data are represented as the mean ± SEM. *p*-values for qRT-PCR analysis and metal ion analysis were determined by using the two-tailed, unpaired Student’s *t*-test and Mann–Whitney test, respectively. Con: Control; Tb: Tumor-bearing; Tum: Tumor cell lines; Gast.: Gastrocnemius muscle.

Building on our previous studies that identified muscle-expressed *Zip14* and zinc imbalances as critical mediators of cachexia in metastatic colon, breast, and lung cancers [21], we examined whether the *Zip14*–zinc axis is also important for PDAC-associated cachexia. We analyzed gastrocnemius muscles from mice bearing either Pan02- or FC1242-derived metastases, as well as age-matched control mice without injection of tumor cells. Consistent with our previous findings [21], there was a robust increase in muscle-expressed *Zip14* at both RNA and protein levels in Pan02 and FC1242 PDAC models, as indicated by quantitative RT-PCR (Figure 2A) and immunoblot analyses (Figure S1A,B), respectively.

ZIP14 is a broad-spectrum transporter of several metal ions, including zinc (Zn^2+^), iron (Fe^2+^), and manganese (Mn^2+^) [23]. Therefore, we analyzed the levels of each of these ions in gastrocnemius muscles by inductively coupled plasma mass spectrometry (ICP–MS). Our results show increased zinc ion levels in cachectic muscles from both Pan02 (Zn^2+^: *p* = 0.0022) and FC1242 (Zn^2+^: *p* = 0.0159) metastasis models compared to controls, but no significant changes in either iron or copper levels (Figure 2B). Only low manganese levels were detected in muscle tissue, which increased in the cachectic Pan02 model (Mn^2+^: *p* = 0.0159) but not in the FC1242 model (Mn^2+^: *p* = n.s.). In addition to ZIP14, 13 other members of the SLC39/ZIP family are known to regulate intracellular zinc levels. Therefore, we analyzed the expression of the remaining 13 *Zip* transporter genes in cachectic muscles from the Pan02 and FC1242 models (Figure S2). Most of the *Zip* transporter genes were induced significantly in cachectic muscles from the FC1242 model, but only minimally induced in cachectic muscles from the Pan02 model (Figure S2). Thus, *Zip14* was the only zinc influx transporter gene that was significantly induced in cachectic muscles from both models (Figure 2A and Figure S2). Metallothioneins (Mt), such as *Mt1* and *Mt2*, are zinc-inducible genes that encode cysteine-rich metal-binding proteins and serve as markers for elevated intracellular zinc ion concentration in cells [24,25]. In line with *Zip14* induction and increased zinc levels, cachectic muscles showed significant upregulation of both *Mt1* and *Mt2* in the Pan02 (*Mt1*: *p* = 0.0072; *Mt2*: *p* = 0.0016) and FC1242 (*Mt1*: *p* = 0.0070; *Mt2*: *p* = 0.0295) models compared to their respective controls (Figure 2C). In addition to muscles, since the ZIP family of transporters, such as *Zip4,* are also expressed in tumor cell lines and play a role in PDAC tumorigenesis [26], we tested the expression of *Zip4* and other ZIP transporters in the Pan02 and FC1242 tumor cells. *Zip4* was abundantly enriched in the FC1242 but not in the Pan02 tumor cell line (Figure S2). In contrast, low levels of *Zip14* were expressed in the Pan02 and FC1242 tumor cells (Figure 2A). Together, our current findings indicate that both *Zip14* expression and intracellular zinc levels are elevated in cachectic muscles from multiple PDAC models. Therefore, altered zinc homeostasis in muscles could be an underlying feature of PDAC-associated cachexia development.

### 2.3. Clinical Validation of Aberrant ZIP14 Expression in Cachectic Pectoralis Muscles from Metastatic PDAC Patients

**Figure 3:**
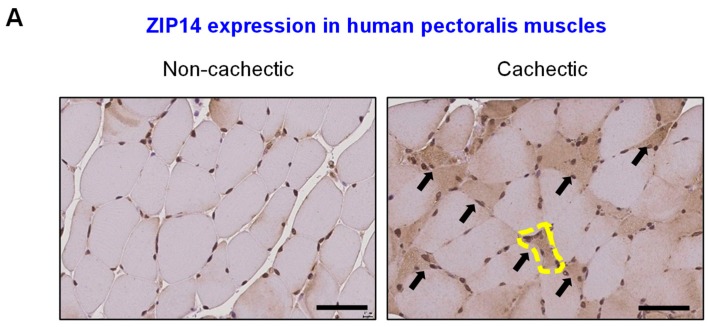

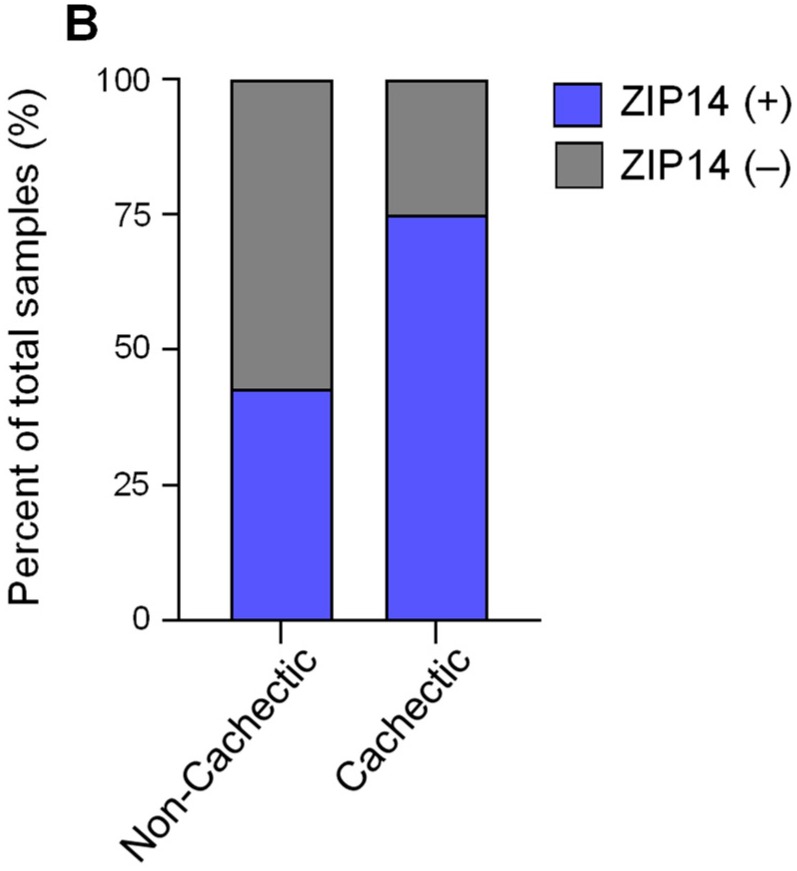
Clinical validation of ZIP14 expression in cachectic pectoralis muscles from metastatic PDAC patients. (**A**) Representative images of ZIP14 immunohistochemical analysis of human pectoralis muscle cross-sections from non-cachectic (left, *n* = 7) and cachectic (right, *n* = 12) pancreatic cancer patients. A representative atrophic fiber is marked by the yellow dotted line. Additional atrophic fibers visualized in the field are marked with arrows. Scale bars represent 50 µm. (**B**) Blinded scoring of ZIP14-stained pectoralis muscle sections as ZIP14-positive (blue bars) or ZIP14-negative (gray bars) from cachectic and non-cachectic cancer patients. Data are shown as percentage of total samples and represented as mean ± SEM. The *p*-value was calculated using Pearson’s chi-square test on scored sample counts (*p* = 0.0005).

To clinically validate our experimental findings associating the ZIP14–zinc axis with PDAC-associated cachexia, we analyzed ZIP14 expression by immunohistochemical analysis of human pectoralis muscle sections from metastatic PDAC patients (Figure 3A).

Blinded pathological analysis showed ZIP14 positivity in the atrophic muscle fibers from 9 of 12 (75%) cachectic PDAC patients compared to 3 of 7 (42.9%) non-cachectic PDAC patients (Pearson’s chi-square test: *p* = 0.0005) (Figure 3A,B). Notably, high ZIP14 expression was observed specifically in the atrophic (compared to non-atrophic) myofibers within the same muscle section from cachectic PDAC patients (Figure 3A). Consistent with our experimental analyses (Figure 1 and Figure 2), these results link ZIP14 expression with muscle atrophy in human PDAC.

### 2.4. Upregulation of Zip14 Expression in Cachectic Diaphragm Muscles in PDAC Models and Patients

**Figure 4:**
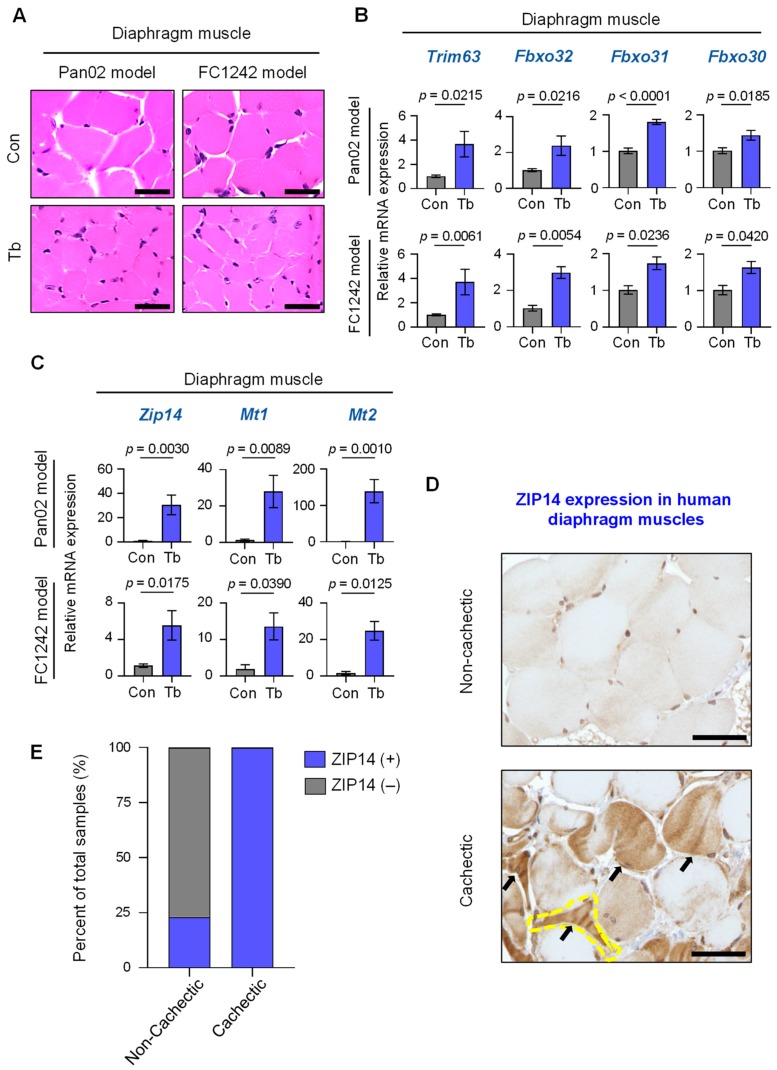
ZIP14 expression in diaphragm muscles from PDAC models and human patients. (**A**) Representative H&E staining of diaphragm muscle cross-sections from Pan02 and FC1242 mice compared to controls. Scale bars represent 25 µm. (**B**) Results from qRT-PCR analysis of *Trim63*, *Fbxo32*, *Fbxo31*, and *Fbxo30* in the diaphragm muscles from Pan02 (top) and FC1242 (bottom) mice (Tb, blue bars) compared to controls (Con, gray bars). (**C**) Results from qRT-PCR analysis of *Zip14*, *Mt1,* and *Mt2* in the gastrocnemius muscles from Pan02 (top) and FC1242 (bottom) mice compared to controls. (**D**) Representative images of ZIP14 immunohistochemical analysis of human diaphragm muscle cross-sections from non-cachectic (top, *n* = 13) and cachectic (bottom, *n* = 10) pancreatic cancer patients. A representative atrophic fiber is marked by the yellow dotted line. Additional atrophic fibers visualized in the field are marked with arrows. Scale bars represent 25 µm. (**E**) Blinded scoring of ZIP14-stained diaphragm muscle sections as ZIP14-positive (blue bars) or ZIP14-negative (gray bars) from non-cachectic and cachectic cancer patients. Data are shown as a percentage of total samples and the *p*-value was calculated using scored sample counts (*p* < 0.0001). *n* = 3–6 mice/group. Data are represented as the mean ± SEM. *p*-values for qRT-PCR analysis were determined using the two-tailed, unpaired Student’s *t*-test. *p*-values for immunohistochemical scoring were determined using the Pearson’s chi-square test. Con: Control; Tb: Tumor-bearing.

To determine whether *Zip14* is systemically upregulated in muscle during cachexia development, we also analyzed the diaphragm for *Zip14* expression and signs of muscle atrophy in the Pan02 and FC1242 PDAC models. The diaphragm is comprised of skeletal muscles that are integrally linked to respiratory function, and dysfunction of the diaphragm muscle can lead to respiratory failures and poor survival in cancer patients [12,27]. Histological examination of diaphragm muscles from the Pan02 and FC1242 PDAC models showed marked reduction in muscle fiber size by hematoxylin and eosin (H&E) staining analysis (Figure 4A). Additionally, the upregulation of muscle atrophy marker genes in the diaphragm muscles of Pan02 (*Trim63*: *p* = 0.0215; *Fbxo32: p* = 0.0216; *Fbxo31*: *p* < 0.0001; *Fbxo30*: *p* = 0.0185) and FC1242 (*Trim63*: *p* = 0.0061; *Fbxo32: p* = 0.0054; *Fbxo31*: *p* = 0.0236; *Fbxo30*: *p* = 0.0420) mice was indicative of diaphragm muscle atrophy (Figure 4B). Similar to gastrocnemius muscles (Figure 2), the expression of *Zip14* and the zinc-inducible genes, *Mt1* and *Mt2,* was significantly increased in cachectic diaphragm muscles compared to controls in the Pan02 (*Zip14*: *p* = 0.0030; *Mt1*: *p* = 0.0089; *Mt2*: *p* = 0.0010) and FC1242 (*Zip14*: *p* = 0.0175; *Mt1*: *p* = 0.0390; *Mt2*: *p* = 0.0125) models (Figure 4C). We next examined ZIP14 expression by immunohistochemical analysis of human diaphragm muscle sections from both metastatic PDAC patients with and without cachexia and patients without cancer or cachexia (Figure 4D). Blinded pathological analysis showed ZIP14 positivity in the atrophic muscle fibers from 10 of 10 (100%) cachectic PDAC patients compared to 3 of 13 (23.1%) non-cachectic patients, which included 4 non-cachectic PDAC patients and 9 non-cachectic non-cancer patients (Pearson’s chi-square test: *p* < 0.0001) (Figure 4E). The increased expression of *Zip14* and zinc-inducible *Mt1* and *Mt2* genes in the diaphragm muscles from the PDAC models and the prominent expression of ZIP14 in atrophic muscle fibers from PDAC patients is suggestive of an aberrant *Zip14*–zinc axis in muscles during PDAC-associated cachexia development.

## 3. Discussion

In line with our previous studies on metastatic colon, breast, and lung cancer cachexia [21], we show in the current study that expression of the zinc transporter, *Zip14,* correlates with increased zinc levels in cachectic muscles in two mouse models of PDAC-associated cachexia, and that ZIP14 is upregulated in the pectoralis and diaphragm muscles from cachectic human PDAC patients. Since our data are derived from a small sample size (7–13 patients per group), future studies that include additional samples are warranted to strengthen the association between ZIP14 expression levels and PDAC-associated cachexia. It will also be important to determine when *Zip14* is induced in muscles during cachexia development and whether a clinical association exists between ZIP14 muscle expression and the stage of PDAC. Our previous studies have shown that TNF-α and TGF-β cytokines can upregulate ZIP14 levels in muscle cells [21]. These cytokines can be detected in the conditioned media of PDAC cell lines and sera of PDAC patients [9,28,29,30,31,32]. It remains to be tested in future studies whether blocking TNF-α or TGF-β signaling in muscles can reduce *Zip14* expression. Moreover, identifying the factors responsible for ZIP14 upregulation in muscles could have important clinical implications. First, if circulating factors in plasma exist that are responsible for mediating muscle-specific *ZIP14* expression, these factors could serve as biomarkers for cachexia development in PDAC patients. Second, if *Zip14* blockade in PDAC models is beneficial and reduces PDAC-associated cachexia, screening for ZIP14 inducers in blood could stratify patients for likelihood of benefit from ZIP14 blockade in the clinic.

Zinc is an important trace element that is critical for maintaining normal cellular physiology. Zinc binds to about 10% of all human proteins through zinc finger motifs, and functions as a cofactor for enzymes and as a secondary messenger [33,34]. In recent studies, *Zip4*, another metal ion transporter that can import zinc into cells, was found to be overexpressed in human pancreatic cancer tissue compared to adjacent normal tissue [26]. We also observed high *Zip4* levels in tumor cells from the FC1242 PDAC model. Li and colleagues found that in PDAC tumor cells, *Zip4* upregulation and the concomitant increase in zinc influx promote tumor growth, and that knockdown of *Zip4* in tumor cells reduces cachexia and prolongs survival in mice [26]. Interestingly, in muscles, *Zip4* mediates the release of extracellular vesicles (EVs) that activate the pro-cachectic p38 MAPK pathway that promotes muscle atrophy [35]. Our current findings are complementary to the findings by the Li group in that both studies underscore the importance of altered zinc homeostasis in the pathogenesis of cachexia in PDAC, albeit through non-overlapping mechanisms.

Metal ion chelation therapy represents another potentially viable strategy to disrupt the *Zip14*–zinc axis. While *Zip14* is robustly expressed in cachectic muscles from both PDAC models, we found that several other *Zip* transporter genes are upregulated in cachectic muscles from the FC1242 model but not in the Pan02 model. Therefore, the inhibition of ZIP14 function or chelation of excess zinc could be tested as a therapeutic strategy to treat PDAC-associated cachexia. A recent study showed that treatment of human PDAC cell lines with the membrane-permeable intracellular zinc chelator, TPEN (N,N,N’,N’-Tetrakis (2-pyridylmethyl)-ethylenediamine), induced tumor cell death by reducing glutathione (GSH) concentrations, increasing oxidative stress, and promoting mitochondrial dysfunction [36]. Moreover, tumor cell death induced by zinc chelators has been reported in leukemia, osteosarcoma, breast, and prostate cancer cell lines in vitro [37,38,39,40,41,42], although in vivo zinc chelation assays in mice have yet to be performed using PDAC models. Studies from Li and colleagues [26,35], our previous studies [21], and our current findings therefore provide a strong rationale for testing the efficacy of zinc chelation strategies and ZIP4/14 blockade in PDAC-associated cachexia.

## 4. Materials and Methods

### 4.1. Mouse Studies

All mouse protocols were approved by The Columbia University Institutional Animal Care and Use Committee (IACUC) and all mouse experiments complied with ethical regulations and guidelines outlined by Columbia University Irving Medical Center’s (CUIMC) Institute of Comparative Medicine (ICM), with the IACUC approval code AAAR6450. Animals were housed within CUIMC’s specific pathogen-free barrier facility with regulated environment and fed Labdiet 5053 (standard diet). Male, 8- to 9-week-old athymic nude mice and C57BL/6 mice were purchased from Envigo (Somerset, NJ, USA) and Jackson Laboratory (Bar Harbor, ME, USA), respectively. Athymic nude and C57BL/6 mice were injected with 1 × 10^5^ murine Pan02 or FC1242 pancreatic cancer cells, respectively, by intra-cardiac injection into arterial circulation. Mouse body condition, as an indicator of cachexia, was evaluated using a body-condition scoring system as previously reported [43]. Cachexia was independently assessed by body weight and muscle strength analysis, histological and morphometric analysis, and molecular marker analysis as outlined in references [21,44].

### 4.2. Measurement of Body Weight and Hind-Limb Grip Strength

Body weights of mice from control and tumor-bearing groups were measured once a week using a bench-top digital balance. Body weights measured on the day of injection (day 0 post-injection) were used to normalize subsequent weight measurements. Body weights were converted to percent change relative to the day 0 measurement. Hind-limb grip strength of mice was measured weekly using a grip strength meter (Columbus Instruments, Columbus, OH, USA). Mean values of hind-limb grip strength for each mouse were determined by replicate readings of a minimum of five measurements performed per time point. Percent grip strength was normalized relative to the mean initial grip strength values (100%) at day 0 post-injection.

### 4.3. Cell Lines

The murine pancreatic cancer cell line Pan02 was derived from a primary pancreatic tumor from a C57BL/6 mouse treated with a carcinogen, 3-methylcholanthrene (3-MCA) [9,22,45]. Pan02 was purchased from the National Cancer Institute Cell Repository, MD, USA. The murine pancreatic cancer cell line FC1242 was generated in the David Tuveson laboratory (Cold Spring Harbor Laboratory, NY, USA) and was isolated and derived from the primary pancreatic tumor from a KPC (Kras^+/LSL-G12D^; Trp53^+/LSL-R172H^; Pdx1-Cre) mouse with a C57BL/6 background [9,46]. FC1242 was obtained from the George Miller laboratory (New York University, NY, USA). The two cell lines were cultured in DMEM supplemented with 10% fetal bovine serum, 100 µg/mL streptomycin, and 100 IU/mL penicillin (Life Technologies, Carlsbad, CA, USA). Cells were grown in a 37 °C CO_2_ incubator (5% CO_2_).

### 4.4. Tissue Collection

Gastrocnemius and diaphragm muscles were collected in 4% paraformaldehyde for histological analysis or snap frozen in liquid nitrogen and stored at −80 °C for further molecular analysis. Liver and lungs were collected from mice and fixed in 4% paraformaldehyde. All samples were fixed for 24 h on a rocker at 4 °C, washed in PBS, and transferred to 70% ethanol until histology and analysis.

### 4.5. Histological Analysis of Metastasis and Muscles

Liver, lung, gastrocnemius, and diaphragm tissues were paraformaldehyde-fixed and paraffin-embedded, and 5 µm sections were stained with hematoxylin and eosin (H&E). H&E-stained liver and lung sections were visualized under a DM5500B microscope (Leica Microsystems, Wetzlar, Germany) for the presence of metastases. H&E-stained gastrocnemius and diaphragm sections were visualized using an Eclipse Ni-U microscope (Nikon Corporation, Tokyo, Japan).

### 4.6. Quantification of Muscle Cross-Sectional Area

Gastrocnemius muscles collected from control and tumor-bearing mice were paraffin-embedded, and 5-µm-thick cross-sections were H&E stained. Random two to three fields per muscle section were imaged at 20× magnification using a DM5500B microscope (Leica Microsystems, Wetzlar, Germany) and analyzed for muscle fiber cross-sectional area quantification (ImageJ software, Version 1.52h, National Institutes of Health, Bethesda, MD, USA). Individual myofiber cross-sectional areas were quantified using a drawing tool in ImageJ to trace fiber boundaries. Myofibers were stratified by cross-sectional area into various size ranges, and the percentage of fibers in each cross-sectional area range was determined for tumor-bearing and control mice. Means of cross-sectional area for each range was determined from replicate muscle samples.

### 4.7. Gene Expression Analysis by qRT-PCR

Gastrocnemius or diaphragm muscles (snap frozen) were cut into ~1–2 mm pieces and a representative sample of 15 mg from each muscle was taken for extraction of total RNA using Trizol (Life Technologies, Carlsbad, CA, USA). Muscle sample lysis was performed using TissueLyser II (Qiagen, Hilden, Germany) and 5 mm steel beads (Qiagen). Supernatants collected after centrifugation (18,000× *g* for 5 min) were processed using the RNeasy Mini Kit (Qiagen) and treated on-column with DNase as per the manufacturer’s instructions (Qiagen). RNA from tumor cells (Pan02 or FC1242) was isolated using the RNeasy Mini Kit (Qiagen) and treated on-column with DNase as per the manufacturer’s instructions (Qiagen). Total RNA was quantified by a Nanodrop spectrophotometer (Thermo Fisher Scientific, Waltham, MA, USA). Purified total RNA (500 ng per sample) was reverse transcribed to cDNA using a cDNA Synthesis Kit (Applied Biosystems (Thermo Fisher Scientific), Carlsbad, CA, USA). qRT-PCR was performed with 10 ng of cDNA per sample using gene-specific primers and the SYBR Green PCR master mix (Applied Biosystems (Thermo Fisher Scientific), Carlsbad, CA, USA). *Gapdh* primers were used as an internal control. Data were analyzed on an Applied Biosystems 7500 Real Time PCR system (Applied Biosystems (Thermo Fisher Scientific), Carlsbad, CA, USA) and gene expression fold change was calculated using the 2^−∆∆Ct^ method [47]. The qRT-PCR primer sequences used in this study are shown separately in Table S1.

### 4.8. Metal Ion Analysis in Muscles

Gastrocnemius muscles (15–20 mg per sample) were sent for metal ion analysis to the Veterinary Diagnostic Laboratory at Michigan State University, MI, USA. Tissues were dried overnight at 75 °C, then subjected to overnight digestion in a 10× volume of nitric acid relative to dry weight of muscle sample and further diluted in a 100-fold volume of water. Metal ion analysis in muscle samples was performed by inductively coupled plasma mass spectrometry (ICP–MS) (Agilent, Santa Clara, CA, USA). A four-point linear curve of analyte–standard response ratio was used to estimate concentrations of elements in muscle samples.

### 4.9. Immunohistochemical Staining of Human Muscle Sections Using ZIP14 Antibody

Human muscle samples were obtained from autopsy cases at the University of Nebraska Medical Center upon approval by the Institutional Review Board (IRB). In brief, pectoralis and diaphragm muscles from cachectic and non-cachectic metastatic PDAC patients were formalin-fixed, paraffin-embedded, and sectioned at 5 µm thickness onto glass slides. Slides were baked at 60 °C for 1 h and de-paraffinized using Histo-clear (National Diagnostics, Atlanta, Georgia, GA, USA), rehydrated, and treated with 1% hydrogen peroxide for 10 min at room temperature. Antigen retrieval was performed in citrate buffer, pH 6.0 (Vector Laboratories, Burlingame, CA, USA) in a steamer apparatus. Endogenous avidin and biotin were blocked using avidin/biotin blocking reagents (Vector Laboratories, Burlingame, CA, USA) at room temperature. All further incubations were performed at room temperature. Slides were further blocked with 2% BSA in PBS containing 10% goat serum for 30 min. Tissue sections were incubated with rabbit polyclonal primary antibody against human ZIP14 (developed in our laboratory as indicated in [21]) (1:1000), followed by biotinylated goat anti-rabbit IgG secondary antibody (BA-1000, Vector Laboratories, Burlingame, CA, USA) (1:250). The ABC kit and DAB kit (Vector Laboratories, Burlingame, CA, USA) were used for amplification of signal and detection of ZIP14 expression, respectively, following the manufacturer’s instructions. Slides were washed three times with PBS after each incubation step during staining. Sections were counterstained with hematoxylin, dehydrated, and mounted using Cytoseal XYL (Richard-Allan Scientific, Kalamazoo, MI, USA) for histological analysis. ZIP14-stained muscles from cachectic or non-cachectic patient samples were scored as ZIP14-positive or -negative staining in a blinded pathological analysis.

### 4.10. Immunoblot Analysis of Muscle Samples

Gastrocnemius muscle samples were collected from Pan02 and FC1242 tumor-bearing and respective control mice. Muscle samples (15 mg) were homogenized in 250 µL of lysis buffer consisting of 150 mM NaCl, 1% NP-40, 0.5% sodium deoxycholate, 0.1% SDS, and 50 mM Tris pH 8.0 supplemented with protease inhibitor (Roche, Mannheim, Germany) and phosphatase inhibitor (Thermo Scientific (Thermo Fisher Scientific), Waltham, MA, USA). Muscles were lysed using the TissueLyser II (Qiagen) for 3 min × 2 cycles and lysate supernatants were collected after centrifugation at 15,000× *g* for 15 min. Protein content was determined by BCA protein assay (Pierce (Thermo Fisher Scientific), Carlsbad, CA, USA). Proteins were transferred to nitrocellulose membranes following SDS-PAGE and membranes were blocked for 1 h with 5% non-fat milk. Membranes were incubated with a rabbit antibody against ZIP14 (1:500) [21], followed by secondary goat anti-rabbit IgG and tertiary donkey anti-goat IgG HRP-conjugated antibodies (Sigma, St. Louis, MO, USA). The membranes were also incubated with an anti-GAPDH rabbit antibody (#5174, Cell Signaling, Beverly, MA, USA) (1:1000), followed by an anti-rabbit IgG HRP-conjugated antibody (Sigma, St. Louis, MO, USA) to probe for GAPDH as a loading control of muscle lysates. The membranes were developed using an ECL substrate (Bio-Rad, Hercules, CA, USA) and were visualized on a Bio-Rad ChemiDoc Imaging System (Bio-Rad, Hercules, CA, USA).

### 4.11. Statistical Analysis

Statistical analyses for body weight, grip strength, myofiber cross-sectional areas, and gene expression were performed by the unpaired, two-tailed Student’s *t*-test using GraphPad Prism 8 (GraphPad Software, San Diego, CA, USA). Statistical analysis for metal ion levels was performed using the Mann–Whitney test using GraphPad Prism 8. Statistical analysis for ZIP14 expression in human muscle samples was performed using Pearson’s chi-square test. All values are shown as mean ± standard error of the mean (SEM). *p*-values < 0.05 were considered statistically significant.

### 4.12. Human Specimens

The Rapid Autopsy Program (RAP) operates in compliance with UNMC IRB 091-01, which requires donor and NOK consent for collection and use of samples in research. The UNMC Rapid Autopsy Program is not considered Human Subjects Research and is exempt under 45 CFR 46.101(b)(4) from 45 CFR part 46 requirements. Human pancreatic cancer, metastatic lesions, pectoralis and diaphragm skeletal muscles, and unaffected specimens from descendants who were previously diagnosed with pancreatic carcinoma were obtained prospectively from the University of Nebraska Medical Center’s (UNMC) Tissue Bank through the Rapid Autopsy Program for Pancreas. Clinical data were obtained for each donor from multiple sources, including the UNMC Pathology autopsy report and patient clinical records accessible through OneChart. Specifically, cachexia status was determined by pathology at the time of death and prior to autopsy. Height, weight, and all other observable donor characteristics were documented by pathology and provided to the RAP program via the autopsy report. All samples and clinicopathological data were deidentified prior to distribution (Table S2).

Pancreatic, duodenal, splenic, liver, and diaphragm specimens from humans not diagnosed with cancer (non-cancer donors) were obtained through collaborative association with Live On Nebraska. All specimens were de-identified prior to distribution to UNMC and were housed under the auspices of the Rapid Autopsy Program. Limited de-identified clinical information was provided, including height and weight (Table S2).

To ensure specimen quality, organs, and tissues were harvested within 3 hours postmortem and the specimens flash frozen in liquid nitrogen or placed in formalin for immediate fixation. Then, 5-µm-thick sections were cut from paraffin blocks of formalin-fixed tissue and mounted onto charged slides for distribution to users.

## 5. Conclusions

Cachexia is a debilitating muscle wasting disorder observed in over 85% of PDAC patients, and is a major contributor to poor quality of life, reduced tolerance to anti-neoplastic therapies, and decreased survival [12,13,48]. Cachectic patients exhibit a significant loss of skeletal and cardiac muscle mass, a function that contributes to their accelerated death from cardiac and respiratory muscle wasting [16,18]; however, there are currently no approved treatments for cachexia. Based on our previous studies identifying a crucial role for the metal ion transporter, ZIP14, in cachexia associated with colon, breast, and lung cancers, we examined the importance of the *Zip14*–zinc axis in PDAC-associated cachexia. Using independent mouse models of PDAC metastasis, we showed that *Zip14* upregulation and altered zinc homeostasis are also underlying features of PDAC-associated cachexia. These findings are relevant to human PDAC-associated cachexia, since aberrant ZIP14 expression in atrophic muscle fibers was significantly correlated with the presence of cachexia in human PDAC patients. Future preclinical studies will ultimately be necessary to determine whether ZIP14 blockade or zinc chelation can serve as viable strategies for preventing or reversing PDAC-associated cachexia.

## Supplementary Materials

The following are available online at https://www.mdpi.com/2072-6694/12/1/3/s1, Figure S1: Immunoblot analysis of ZIP14 in gastrocnemius muscles in PDAC models, Figure S2: Expression of *Zip* genes *(1–13)* in the gastrocnemius muscles from Pan02, FC1242 tumor-bearing and control mice, and tumor cell lines (Pan02 and FC1242) by qRT-PCR analysis, Table S1: Primers used for qRT-PCR analysis, Table S2: RAP De-identified human patient information.

## Abbreviations

Ath	Athymic nude mice
B6	C57BL/6 mice
Con	Control
CSA	Cross-sectional area
Cu^2+^	Copper
EV	Extracellular vesicles
Fbxo30	F-box protein 30, MUSA1
Fbxo31	F-box protein 31
Fbxo32	F-box protein 32, Atrogin-1
Fe^2+^	Iron
Gast	Gastrocnemius muscle
H&E	Hematoxylin and eosin
ICP–MS	Inductively coupled plasma mass spectrometry
Mn^2+^	Manganese
Mt1	Metallothionein 1
Mt2	Metallothionein 2
PDAC	Pancreatic ductal adenocarcinoma
p38 MAPK	p38 mitogen-activated protein kinase
SLC39A14	Solute carrier family 39 member 14,
Tb	Tumor-bearing
TGF-β	Transforming Growth Factor-beta
TNF-α	Tumor Necrosis Factor-alpha
TPEN	N,N,N′,N′-Tetrakis (2-pyridylmethyl)-ethylenediamine
Trim63	Tripartite motif containing 63, MuRF1
Tum	Tumor cell line
ZIP4	ZRT- and IRT-like protein 4
ZIP14	ZRT- and IRT-like protein 14
Zn^2+^	Zinc
