# Supplementary material for: Upregulation of ZIP14 and Altered Zinc Homeostasis in Muscles in Pancreatic Cancer Cachexia
Source: Cancers (Basel). 2019 Dec 18;12(1):3. doi: 10.3390/cancers12010003 (PMC7016633; doi:10.3390/cancers12010003)
Supplement: Supplementary file 1 [file cancers-12-00003-s001.pdf]

cancers

MDPI

Supplementary Materials: Upregulation of ZIP14 and Altered Zinc Homeostasis in Muscles in Pancreatic Cancer Cachexia

Ahmad Rushdi Shakri, Timothy James Zhong, Wanchao Ma, Courtney Coker, Sean Kim, Stephanie Calluori, Hanna Scholze, Matthias Szabolcs, Thomas Caffrey, Paul M. Grandgenett, Michael A. Hollingsworth, Kurenai Tanji, Michael D. Kluger, George Miller, Anup Kumar Biswas and Swarnali Acharyya

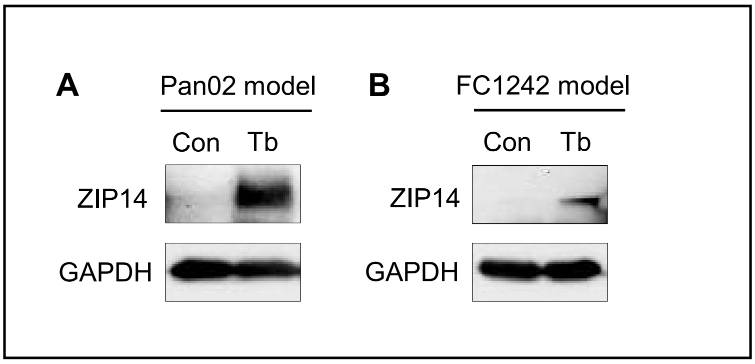

Figure S1. Immunoblot analysis of ZIP14 in gastrocnemius muscles in PDAC models. Representative immunoblots of ZIP14 expression in gastrocnemius muscle from Pan02 tumor-bearing and respective control **(A)**, and FC1242 tumor-bearing and respective control **(B)** mice. GAPDH was used as a loading control. Control is designated as Con and Tumor-bearing as Tb.

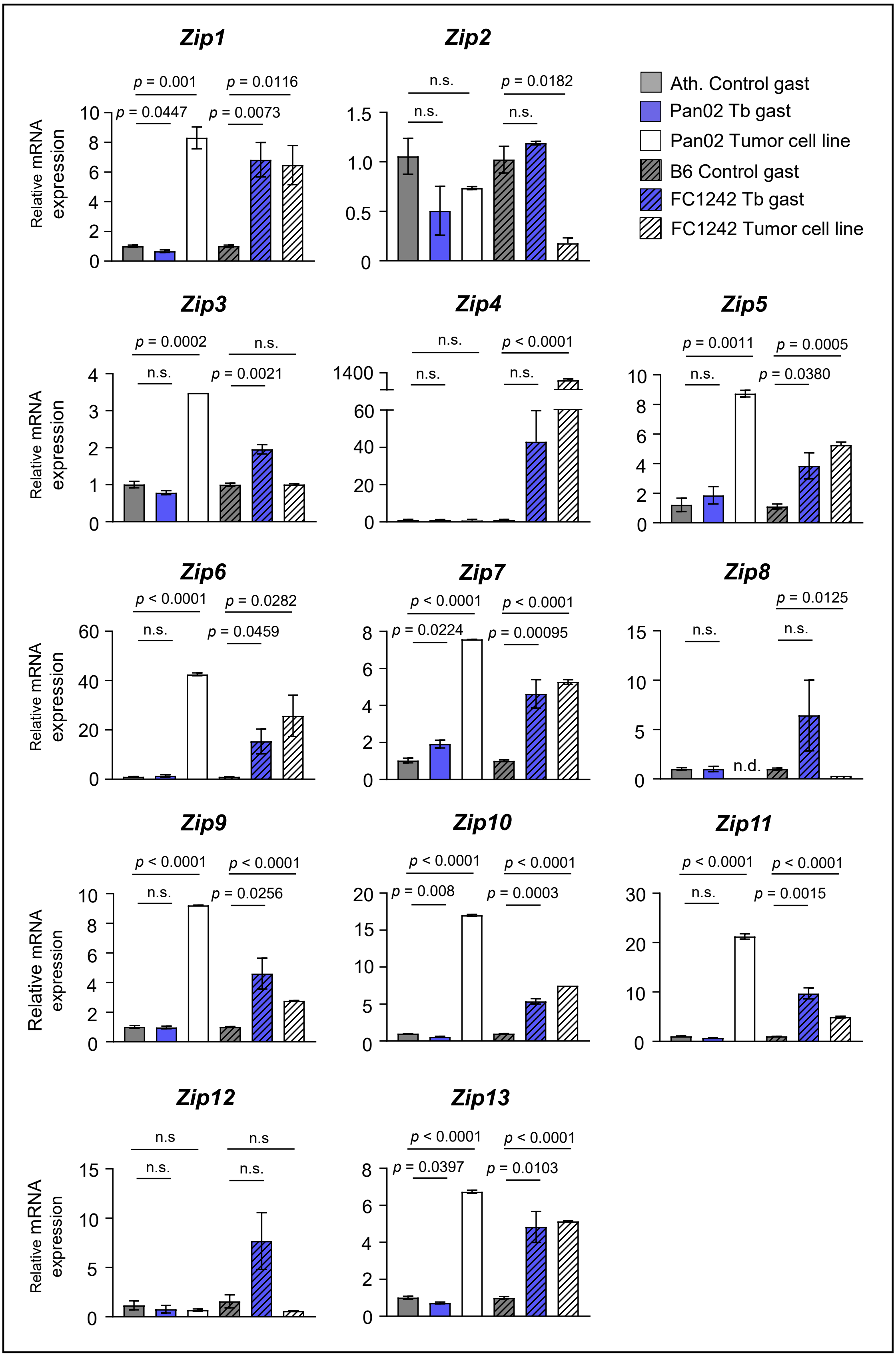

Figure S2. Expression of *Zip* genes (*1–13*) in the gastrocnemius muscles from Pan02, FC1242 tumor-bearing and control mice, and tumor cell lines (Pan02 and FC1242) by qRT-PCR analysis. $n = 3$ mice per group and $n = 2$ replicates for tumor cell lines. Data is expressed as mean ± SEM. p-values were determined by the two-tailed, unpaired Student's t-test. Gray bars: gastrocnemius from athymic non-tumor bearing control mice; blue bars: gastrocnemius from Pan02 tumor bearing mice; white bars:

Pan02 tumor cell line; shaded gray bars: gastrocnemius from C57BL/6 non-tumor bearing control mice; shaded blue bars: gastrocnemius from FC1242 tumor-bearing mice; shaded white bars: FC1242 tumor cell line. n.s. not significant; n.d.: not detected; Ath.: athymic nude mice; B6: C57BL/6 mice; Tb: tumor-bearing; gast: gastrocnemius muscle.

Table S1. Sequences of primers used in the study for expression analysis of mouse *Zip1-14*, *Trim63/MuRF1*, *Fbxo32/MAFBx*, *Fbxo31*, *Fbxo30/MUSA1*, *Mt1*, *Mt2*, and *Gapdh*.

Name of Gene	Forward Primer (5′–3′)	Reverse Primer (5′–3′)
Mouse-*Zip1*	AGGTCAGGTGCTAACCATGAA	CTGTTCCTTGTAAGCCAGCGT
Mouse-*Zip2*	GGTTCCAGATGGATGCAGCTA	CTCCACGTAACTAGAAGCAGC
Mouse-*Zip3*	GGTGGCGTATTCCTGGCTAC	CTGCTCCACGAACACAGTGA
Mouse-*Zip4*	ATGCTCCCAAAGTCGGTCAC	CAGCGTATTTAACAGGCCGTC
Mouse-*Zip5*	ATCATCTGCTGACTGGCCTAT	CAGTGTCCCGTTCTCTCCATA
Mouse-*Zip6*	GTCACACGGTTGCTGGTAAAA	GGGCGAGATCCTTTCCCTAGA
Mouse-*Zip7*	TGAAAGCATCTGGCATGGG	TGGAGGCTATCGTGGGAGTG
Mouse-*Zip8*	GCCAAGCTCATGTACCTGTCT	AAGATGCCCCAATCGCCAA
Mouse-*Zip9*	TGTTGGTGGGATGTTACGTGG	GATGACCGCCAGTGCAGTT
Mouse-*Zip10*	TCATCGCCATCGTTTGCATCA	CTCTGGTGAAGGGCTGTGAC
Mouse-*Zip11*	CTCCAAGGTTACAGCTCCGTG	CCAGGCTTCCGTCTAAGATCC
Mouse-*Zip12*	GAAGCAGATGCACTGTTACTCA	GCCAAGATGTCATCAGTCTCCT
Mouse-*Zip13*	AGTGGCTATCTCAACCTGCTT	GCCCGATCTTTTTGCTCACAA
Mouse-*Zip14*	GTGTCTCACTGATTAACCTGGC	AGAGCAGCGTTCCAATGGAC
Mouse-*Trim63/MuRF1*	GTGTGAGGTGCCTACTTGCTC	GCTCAGTCTTCTGTCCTTGGA
Mouse- *Fbxo32/MAFbx*	CAGCTTCGTGAGCGACCTC	GGCAGTCGAGAAGTCCAGTC
Mouse-*Fbxo31*	CATGCGGTTCAAGCCACTG	GTCTGGTTACACTTGGTGGAG
Mouse-*Fbxo30/MUSA1*	TCGTGGAATGGTAATCTTGC	CCTCCCGTTTCTCTATCACG
Mouse -*Mt1*	AAGAGTGAGTTGGGACACCTT	CGAGACAATACAATGGCCTCC
Mouse -*Mt2*	GCCTGCAAATGCAAACAATGC	AGCTGCACTTGTCGGAAGC
Mouse -*Gapdh*	AGGTCGGTGTGAACGGATTTG	TGTAGACCATGTAGTTGAGGTCA

Table S2. De-identified human patient information. (**A**) Rapid Autopsy Program (RAP) patient numbers: cachectic and non-cachectic pancreatic cancer patients. (**B**) RAP NORS 1, 3, 13, 21, 23, 24, 25, 28 and 32: non-cachectic, non-cancer patients.

(**A**)

RAP #	Age	Sex	Survival Days	Stage at Diagnosis	Stage at Endpoint Collection	Cachexia (1) Presence of Cachexia: 1 and Absence: 0
105	89	F	26	IV	IV	0
107	83	F	269	IV	IV	1
108	78	M	762	IIA	IV	1
109	64	M	405	IV	IV	1
110	36	M	138	IV	IV	1
111	43	M	326	IV	IV	0
112	84	F	21	IV	IV	0
113	58	F	878	IV	IV	1
115	80	M	63	IV	IV	0
116	80	F	497	IV	IV	1
117	68	M	316	III	IV	1
118	72	M	38	IV	IV	0
119	85	F	183	IV	IV	1
120	69	M	507	III	IV	1
121	75	F	235	III	IV	0
123	74	M	90	IV	IV	0
124	53	F	421	IV	IV	1
125	49	F	27	IV	IV	1
126	60	F	353	III	IV	1

(B) RAP Non-cancer controls

RAP NORS ID	Age	Sex	Height (cm)	Weight (kg)
1	59	M	194	93
3	58	F	164	99
13	61	M	170	89
21	53	M	180	110
23	39	M	172	121
24	48	F	165	66
25	36	F	156	70
28	57	F	155	66
32	48	M	163	67

© 2019 by the authors. Licensee MDPI, Basel, Switzerland. This article is an open access article distributed under the terms and conditions of the Creative Commons Attribution (CC BY) license (http://creativecommons.org/licenses/by/4.0/).
